# Enabling Low-Dose In Vivo Benchtop X-ray Fluorescence Computed Tomography through Deep-Learning-Based Denoising

**DOI:** 10.3390/jimaging10060127

**Published:** 2024-05-22

**Authors:** Naghmeh Mahmoodian, Mohammad Rezapourian, Asim Abdulsamad Inamdar, Kunal Kumar, Melanie Fachet, Christoph Hoeschen

**Affiliations:** Chair of Medical Systems Technology, Institute for Medical Technology, Faculty of Electrical Engineering and Information Technology, Otto von Guericke University, 39106 Magdeburg, Germany; mohammad.rezapourianghahfarokhi@ovgu.de (M.R.); asim.inamdar@st.ovgu.de (A.A.I.); kunal.kumar@ovgu.de (K.K.); melanie.fachet@ovgu.de (M.F.); christoph.hoeschen@ovgu.de (C.H.)

**Keywords:** deep learning (DL), artificial intelligence (AI), X-ray fluorescence (XRF), XFCT, nanoparticles, cancer

## Abstract

X-ray Fluorescence Computed Tomography (XFCT) is an emerging non-invasive imaging technique providing high-resolution molecular-level data. However, increased sensitivity with current benchtop X-ray sources comes at the cost of high radiation exposure. Artificial Intelligence (AI), particularly deep learning (DL), has revolutionized medical imaging by delivering high-quality images in the presence of noise. In XFCT, traditional methods rely on complex algorithms for background noise reduction, but AI holds promise in addressing high-dose concerns. We present an optimized Swin-Conv-UNet (SCUNet) model for background noise reduction in X-ray fluorescence (XRF) images at low tracer concentrations. Our method’s effectiveness is evaluated against higher-dose images, while various denoising techniques exist for X-ray and computed tomography (CT) techniques, only a few address XFCT. The DL model is trained and assessed using augmented data, focusing on background noise reduction. Image quality is measured using peak signal-to-noise ratio (PSNR) and structural similarity index (SSIM), comparing outcomes with 100% X-ray-dose images. Results demonstrate that the proposed algorithm yields high-quality images from low-dose inputs, with maximum PSNR of 39.05 and SSIM of 0.86. The model outperforms block-matching and 3D filtering (BM3D), block-matching and 4D filtering (BM4D), non-local means (NLM), denoising convolutional neural network (DnCNN), and SCUNet in both visual inspection and quantitative analysis, particularly in high-noise scenarios. This indicates the potential of AI, specifically the SCUNet model, in significantly improving XFCT imaging by mitigating the trade-off between sensitivity and radiation exposure.

## 1. Introduction

In recent times, X-ray fluorescence (XRF) imaging has garnered significant interest as a promising tool for in vivo preclinical studies due to its ability to quantify and determine the biodistribution of labeled nanoparticle-based contrast agents at high resolution [1,2,3,4]. For this purpose, various imaging approaches have been proposed with different scanning methodologies and X-ray sources for the excitation of characteristic X-ray photons from specific metallic nanoparticles [1,5]. Some studies have measured the distribution of nanoparticles in mice using monochromatic X-rays from synchrotron sources [6,7,8,9]. Meanwhile, other studies have applied conventional X-ray tubes with polychromatic X-rays for specific biomedical applications at the laboratory scale [3,4,10,11,12,13,14,15,16,17,18]. To name a few, Cong et al. [16] effectively reconstructed gold nanoparticles by employing a fan-beam X-ray source and parallel single-hole collimation. Deng et al. [12] employed a conventional X-ray tube to observe the distribution of gadolinium nanoparticles within mouse kidneys. Specifically, X-ray fluorescence computed tomography (XFCT), which leverages the principles of XRF imaging within a computed tomography (CT) framework, has been studied in several previous works [10,11,15,17,18].

In both approaches, the primary source of background noise is the appearance of Compton-scattered photons in the signal region, causing a low signal-to-noise ratio and reduced detection sensitivity. Contributions from Rayleigh scattering are, typically, minimal due to predominant scattering in forward directions, away from the detectors. Hence, enhancing image quality involves the essential task of minimizing Compton background noise to solve the mentioned problem. Approaches available for extracting XRF signals from Compton-scattered photons, including minimization of the latter, involve, e.g., special background-reduction schemes through spatial filtering algorithms and subtraction techniques, among others [3,9,15,17,18]. Specifically, subtraction techniques require consistency in the positioning and posture of the measured object, necessitating two scans (with and without contrast agents, such as nanoparticles) [13,14,19]. This may increase radiation dose, potentially leading to detrimental effects, such as radiation-induced cancer and metabolic abnormalities, among others [19]. Meanwhile, other methods can be cumbersome, may require highly specialized operational knowledge, or require significant user intervention. This motivates a shift from traditional noise suppression techniques to new AI-based approaches, like deep learning denoising, offering promising advantages and moving towards automation.

Recently, deep learning denoising methods have been widely used in the field of biomedical imaging and image processing [20,21,22,23]. The convolutional neural network (CNN) architecture can extract features from low to high levels, resulting in better identification and understanding of patterns [24]. The methods show a high performance of image-to-image translation issues [25,26]. For example, achieving a high-quality (high-dose) image from a low-dose image by removing image noise [25]. In contrast to the assumption made in non-blind image denoising, which presupposes knowledge of the image noise type and level, blind denoising addresses scenarios in which either the noise level, the specific noise type, or both are unknown. Zhang et al. [26] show that a deep model is capable of managing Gaussian denoising across different noise levels. Meanwhile, Chen et al. [27] suggest employing generative adversarial networks (GAN) for noise modeling from clean images. The generated noise is then used to create paired training data for subsequent training purposes. Guo et al. [28] introduce a convolutional blind denoising network, which incorporates a subnetwork for estimating noise. They further suggest training the model using both a practical noise model and pairs of real-world noisy–clean images. Krull et al. [29] introduce a method using variational inference for blind image denoising. This method unifies both noise estimation and image denoising within a Bayesian framework. Sun and Tappen [30] introduce a non-local deep learning approach that combines the benefits of block matching and 3D filtering (BM3D) and non-local means (NLM). Furthermore, the conventional BM3D technique is expanded into a four-dimensional space (BM4D), leading to enhanced preservation of image edge and texture intricacies Zhang et al. [31], Xu et al. [32]. Lefkimmiatis [33] creates an unrolled network capable of executing non-local processing, leading to enhanced denoising outcomes. Liang et al. [34] show improvement in peak signal-to-noise ratio (PSNR) performance achieved by a specific method, i.e., image restoration using the Swin transformer (SwinIR), compared to denoising convolutional neural network (DnCNN) on a benchmark dataset. Liu et al. [35] employ a transformer-based method, the shifted window transformer (Swin), as the primary building block. They demonstrate that the Swin model exhibits improved performance when handling images with repetitive structures, verifying the effectiveness of the transformer in enabling non-local modeling capabilities [35]. A further study proposed a new blind denoising network architecture named Swin-Conv-UNet (SCUNet), applied it to a real image dataset, and designed it for improved practical usage and enhanced local and non-local modeling abilities [26].

In this study, our objective is to employ a blind hybrid deep learning model, SCUNet, and enhance its capability for denoising XFCT images. The model is a result of combining the U-net [36] and Swin [26] models, utilizing both local and non-local modeling. These two networks have individually shown promising denoising performance. The XRF images of a Gadolinium (Gd) contrast agent were generated from high-dose and low-dose measurements to estimate the effect of the radiation dose on background noise by applying the blind hybrid denoising method. The model is created to combine two specific abilities, i.e., the local modeling ability of a residual convolutional layer and the non-local modeling ability of a Swin transformer block. This combined block is suggested for use as a fundamental component within the image-to-image translation UNet architecture, potentially enhancing its performance or capabilities. However, this architecture alone does not suffice for attaining optimal results. Consequently, we incorporated a compound loss function to enhance the network’s performance, specifically addressing the intricacies associated with denoising medical images.

## 2. Materials and Methods

### 2.1. Experimental Setup

The data used in this study consists of XRF images acquired in a previous study using small-animal-sized phantoms consisting of contrast-agent-filled target tubes [10]. The following briefly describes the experimental setup and image acquisition procedure for completeness.

**Figure 1:**
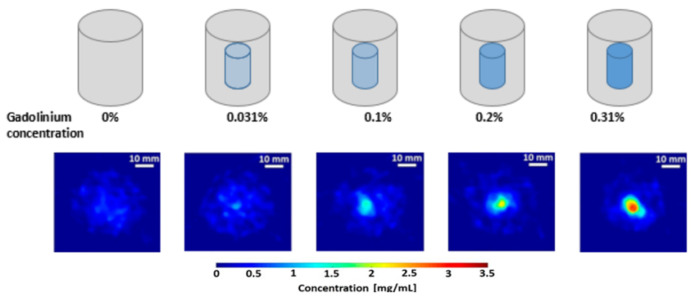
Gadolinium-contrast-filled small-animal phantoms with Gd concentrations ranging from 0 to 3.1 mg/mL.

Gadolinium (Gd) at varying concentrations, derived from Gadoteric acid (C_16_H_25_GdN_4_O_8_), was utilized as the contrast agent. The small-animal-sized surrogate phantoms (mimicking a size-scale on the order of, e.g., mice) consisted of water-filled hexagonal borosilicate glass containers with a maximum diameter of 50 mm and a height of 60 mm. To accommodate 3D breast cancer models immersed in the cell-culture growth medium, the phantoms were chosen with a diameter exceeding that of a typical mouse’s torso (i.e., about 20–25 mm) The Gd–filled polypropylene microcentrifuge tubes, 8 mm in diameter, were embedded inside these water-filled phantoms. Gadolinium (Gd) was used as a contrast agent for various reasons. High atomic number (high-Z) elements, such as Gd (K-alpha emission around 43 keV), exhibit a relatively weaker energy-dependent photon attenuation compared to lower atomic number (low-Z) elements, e.g., iodine (K-alpha emission around 28.4 keV) at this size-scale. Additionally, cadmium telluride (CdTe) pixel detectors were used for the XFCT imaging that exhibits high sensor-intrinsic X-ray fluorescence (i.e., K-shell emission from Cd and Te), making the low energy range (roughly, 19–32 keV) unusable. Consequently, high-Z contrast agents become more favorable at this scale, making Gd a better choice over low-Z elements. These phantoms were used in the previous study to investigate the suitability and feasibility of cone-beam XFCT for both preclinical in vivo imaging of small animals and in vitro surrogate investigations with non-destructive analysis of biological samples [10]. The Gd tubes had concentrations ranging from 0 to 3.1 mg/mL (0, 0.031%, 0.1%, 0.2%, and 0.31% Gd by weight (wt.%), see Figure 1). The first row of Figure 1 shows a representative illustration of the varying Gd concentration, and the second row shows an example of the XFCT scan in the presence of different Gd concentrations.

**Figure 2:**
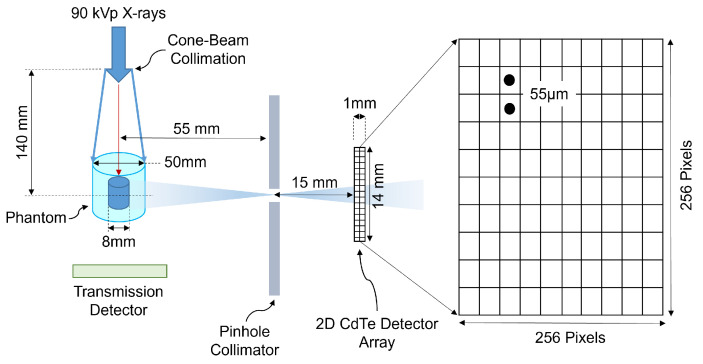
Schematic representation of the experimental benchtop XFCT system.

Attenuation images, acquired using cone-beam computed tomography (CBCT), were used to correct for attenuation in the XRF images. The images were acquired using cone-beam polychromatic X-rays generated from a tungsten-target microfocus X-ray tube (Oxford Nova 600, Oxford Instruments X-ray Technology, Scotts Valley, CA, USA). The incident polychromatic X-rays with 90 kVp, a maximum beam current of 0.9 mA, 0.3 mm copper (Cu) filtration, and X-ray focal spot size of 14–20 μm were used as an excitation source for both CBCT and XFCT imaging. The source-to-isocenter distance was 40.5 cm and the distance from source to transmission detector was 55.7 cm. Figure 2 shows a schematic illustration of the experimental benchtop imaging system, consisting of both CBCT and XFCT imaging configurations. For attenuation images, a total of 30 projections were captured with an angular interval of $12^{\circ }$ between each projection, and approximately 6 s exposure time per projection.

The XFCT imaging system consisted of a Timepix3 HPCD (Minipix TPX3, Advacam, Prague, Czech Republic), with a pixel size of 55 μm, an active detection area of approximately 14 mm × 14 mm, 1 mm thick CdTe sensor, and an energy resolution of around 4–6 keV FWHM at Gd XRF energies. The detector was exposed to radiation using a circular-aperture single-pinhole arrangement. The pinhole collimator was constructed from lead, with an aperture diameter of 0.4 mm and a thickness of 1.5 mm. To conduct full-field scanning within a geometrically/mechanically constrained large CT detector arrangement, the distance between the X-ray source and the rotation isocenter was adjusted to 14 cm. Distances of 5.5 cm from the X-ray source to the isocenter were set, with a pinhole-to-detector distance of 1.5 cm. For XFCT imaging, a sparse-view image acquisition approach was employed to reduce radiation exposure. This strategy involved taking 10 angular projections within a $360^{\circ }$ scan, each with an exposure time of 150 s per projection.

### 2.2. Deep Blind Image Denoising Model

#### 2.2.1. Dataset

To estimate the effect of radiation dose on background noise, images with various noise levels are generated (i.e., corresponding to radiation doses from high to low). The noisy datasets are generated by reducing the photon counts in raw data (i.e., where 100% correspond to high-dose images) by a factor of 25%, 50%, and 75% (corresponding to the low-dose images). Additionally, for each image, three bin widths corresponding to 0.05 keV, 0.1 keV, and 0.5 keV are used. Herein, smaller bin widths correspond to a finer sampling grid, however, with a reduced number of entries per XRF signal bin. Furthermore, to address the limitation posed by the small dataset, we employed a data augmentation technique known as rotation. Data augmentation involves creating additional training samples by applying various transformations to the existing data. While augmentation techniques like rotation, flipping, or scaling increase the dataset size, the generated images primarily remain highly correlated with the original images. They have not been seen to introduce truly new anatomical variations or disease presentations. DL models generally perform better when they are trained on a diverse set of data that reflects the real-world variations that would be encountered during deployment. However, geometric augmentations may not fully provide this diversity. Rotating medical images can disrupt the correct anatomical orientation of organs and structures. This can make interpretation difficult or misleading for clinicians. Additionally, if the image labels are not adjusted after rotation, the model may learn incorrect associations between features and labels, leading to errors. Furthermore, certain imaging modalities might be more sensitive to rotations than others due to their specific acquisition and representation of data. However, it must be noted that these aspects cannot be fully investigated currently as the XFCT technique is only at a nascent stage. More data would be required to fully understand the impact of these augmentations.

In our case, rotation augmentation was chosen to enhance the diversity of the dataset. Rotation augmentation entails rotating the original images by certain angles to generate new perspectives and variations. We specifically chose rotation angles of $90^{\circ }$, $180^{\circ }$, and $270^{\circ }$ for several reasons. Firstly, these angles represent orthogonal transformations, allowing the model to learn diverse features and patterns that may be present in different orientations. Secondly, these angles align with common geometric transformations, capturing potential variations that could be encountered in real-world scenarios. The SCUNet training pipeline involves utilizing denoised images as targets and noisy images as input for the model. Through this approach, the sample size was effectively increased by approximately tenfold. Within the training pipeline, a hold-out strategy was implemented, allocating 20% of the samples for testing in each category and utilizing the remaining 80% for training purposes. The DL model is trained using augmented data and evaluated for background noise reduction via the proposed denoising approach.

#### 2.2.2. Proposed Model

One of the key challenges currently in X-ray fluorescence imaging, particularly when using benchtop/clinical-grade X-ray sources, is the high radiation exposure required to reach high sensitivity. This currently hinders the broader adoption of this technique for in vivo preclinical studies and its translation into future clinical applications. In this study, a blind denoising method was employed to remove noise present in the low-dose images. In blind image denoising, the process of obtaining an estimated clean image involves solving a specific Maximum A Posteriori (MAP) problem using an optimization algorithm. This allows us to estimate the original (clean) image X from noisy observations, a critical step in enhancing XFCT image quality, by employing the following optimization technique:

(1) $$X=\underset{x}{\mathrm{argmin}}\;D\left(x,y\right)+\lambda P\left(x\right),$$

where D(x,y) represents the data fidelity component, P(x) represents the prior term, and λ is the trade-off parameter [26]. At this point, it becomes evident that the crux of addressing blind denoising is twofold: modeling the degradation process of a noisy image and designing the image prior to a clean image. Conceptually, by regarding the deep model as a condensed unrolled inference of Equation (1), the overarching objective of deep blind denoising typically involves tackling the following bi-level optimization problem [37,38]. The effectiveness of the deep blind learning model is largely dependent on its network architecture and the quality of the training data. The presence of noisy images in the training dataset significantly influences the model’s understanding of the degradation process. Improvements in the network architecture and the inclusion of clean images within the training dataset play a crucial role in shaping this understanding. Improving the quality of clean data is achievable, but additional investigation is necessary to enhance and develop network architecture.

**Figure 3:**
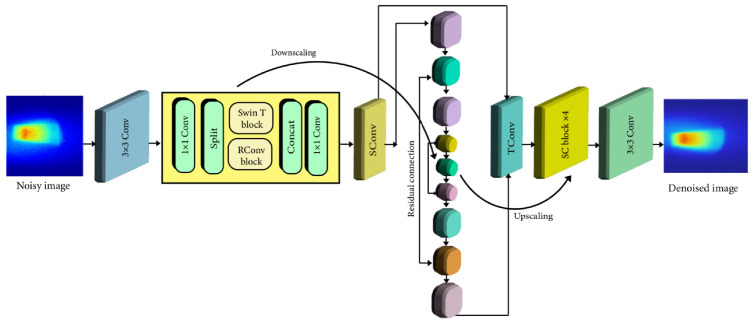
Architecture of the proposed denoising network method.

The network of the SCUNet model is shown in Figure 3. SCUNet (Spatial and Channel-wise Attention U-Net) specifically incorporates attention mechanisms to identify and preserve relevant image features while suppressing noise effectively. This is highly beneficial for the complex data generated by XFCT. The U-Net architecture, integrated within SCUNet, is particularly adept at medical image segmentation and denoising tasks. Its ability to analyze information at multiple scales ensures the preservation of both fine details and broader image context. By integrating the power of deep learning with the specific advantages of SCUNet, our study aims to provide a crucial solution for reducing radiation exposure in XFCT. This has the potential to enhance XFCT imaging, moving towards complete solutions that could enable its broader adoption into research and future medical applications. The core concept behind SCUNet involves merging the complementary network architecture designs from dilated-residual U-Net (DRUNet) and SwinIR. The central technical contribution of our study lies in the development of the optimized SCUNet model. This hybrid architecture uniquely combines the strengths of Convolutional Neural Networks (CNNs), and transformer-based models, Specifically, SCUNet incorporates novel swin-conv (SC) blocks into a UNet backbone [36]. The novel SC block is the heart of SCUNet. It integrates a Swin transformer for non-local modeling with a residual convolutional block for local modeling. This combination allows the model to effectively capture complex image features and noise patterns for superior denoising. The SC blocks are integrated into a multiscale UNet architecture. This enables the model to process information at varying resolutions, enhancing its ability to preserve fine details while suppressing noise across different scales. The hybrid design of SCUNet suggests that it may be more adaptable to the diverse noise characteristics present in XFCT data compared to models relying solely on convolutional or transformer-based architectures. The use of 1 × 1 convolutions within the SC block facilitates seamless information exchange between the transformer and convolutional components, likely improving denoising performance. In accordance with DRUNet [39], SCUNet’s UNet backbone consists of four scales, each featuring a residual connection between 2 × 2 strided convolution (SConv)-based downscaling and 2 × 2 transposed convolution (TConv)-based upscaling. The channel counts in each layer vary from 64 in the first scale to 512 in the fourth scale. A key distinction between SCUNet and DRUNet lies in the adoption of four SC blocks, as opposed to four residual convolution blocks, in each scale of the downscaling and upscaling processes. The images of the XRF photons including Compton scattered photons were inputted into the network with a size of 256 × 256 pixels, and the output images were all the same size. The transformer layer SwinIR model consists of shallow feature extraction, deep feature extraction, and high-quality image reconstruction. We treat each patch as a token and 2 × 2 strided convolution with stride 2. The UNet backbone of our model has four scales, each of which has a residual connection between 2 × 2 SConv-based downscaling and TConv-based upscaling. The number of channels in each layer, from the first scale to the fourth scale, is 64, 128, 256, and 512, respectively.

The second box in Figure 3 illustrates an SC block that combines a Swin transformer (SwinT) block [35] with a residual convolutional (RConv) block [40,41] through two 1 × 1 convolutions, split and concatenation operations, and a residual connection. Specifically, for an input feature tensor, it undergoes an initial 1 × 1 convolution. Following this, the tensor is evenly divided into two feature map groups, namely X1 and X2. This entire process can be expressed as follows:

(2) $$X1,X2=\mathrm{Split}({\mathrm{Conv}}_{1\;\times \;1}\left(X\right))$$

Subsequently, X1 and X2 are individually inputted into a SwinT block and an RConv block, leading to:

(3) Y1,Y2=SwinT(X1),RConv(X2)

Finally, Y1 and Y2 are concatenated to form the input of a 1 × 1 convolution, which establishes a residual connection with the initial input tensor, X. Consequently, the ultimate output of the SC block is expressed as:

(4) $$Z={\mathrm{Conv}}_{1\;\times \;1}\left(\mathrm{Concat}\left(Y1,Y2\right)\right)+X$$

Our proposed SCUNet exhibits several advantages attributed to its innovative module designs. Firstly, the SC block integrates the local modeling capability of the RConv block with the non-local modeling ability of the SwinT block. Secondly, the local and non-local modeling capabilities of SCUNet are further enhanced through the incorporation of a multiscale UNet. Thirdly, the 1 × 1 convolution plays a crucial role in effectively and efficiently facilitating information fusion between the SwinT block and the RConv block. Fourthly, the split and concatenation operations serve as a form of group convolution with two groups, contributing to the reduction in computational complexity and the number of parameters. The parameters are optimized by minimizing the L1 loss with the Adam optimizer [42]. The learning rate starts from $1\times 10^{-4}$ and decays by a factor of 0.9 for each iteration for 40 epochs. The batch size is set to 2. We first train the model with a 25% noise level and then fine-tune the model for other noise levels. All experiments are implemented using PyTorch 2.0.1. It takes about 20 h to train a denoising model on an NVIDIA GTX 1080.

It is noteworthy that SCUNet operates as a hybrid CNN–Transformer network, integrating features from both architectures. Similar approaches exist in the literature, where researchers have explored the combination of CNNs and Transformers for effective network architecture design. It is significant to highlight the distinctions between our proposed SCUNet and two recent works, namely Uformer [43] and Swin-Unet [44].

Firstly, the motivation behind each approach differs significantly. SCUNet is inspired by the observation that state-of-the-art denoising methods, DRUNet [39] and SwinIR [34], leverage distinct network architecture designs. As a result, SCUNet seeks to integrate the complementary features of DRUNet and SwinIR. Conversely, Uformer and Swin-UNet aim to merge transformer variants with UNet, serving a different motivation.

Secondly, the primary building blocks employed in each model are distinct. SCUNet incorporates a novel swin-conv block, which integrates the local modeling capability of the residual convolutional layer [40] with the non-local modeling ability of the Swin transformer block [35] through 1 × 1 convolution, split, and concatenation operations. In contrast, Uformer adopts a novel transformer block by combining depth-wise convolution layers [45], while Swin-UNet utilizes the Swin transformer block as its primary building block.

In this research two evaluation metrics, i.e., peak signal-to-noise ratio (PSNR) and structural similarity index (SSIM), are utilized as they offer advantages and better performance over traditional metrics like Mean Squared Error (MSE) in perceptual quality [46]. Specifically, SSIM, for its feature and structural measures, has been used in several application areas such as denoising, pattern recognition, image restoration, image compression, and more [46]. Both PSNR and SSIM, have shown to perform well in predicting and reflecting the visual quality of images [46].

## 3. Results

As outlined in Section 2.2, enhancing the performance of the deep blind denoising model relies on the optimization of both the network architecture and the training data. In this section, we evaluate the performance of our model qualitatively and quantitatively and compare it with other deep and non-deep learning methods.

**Figure 4:**
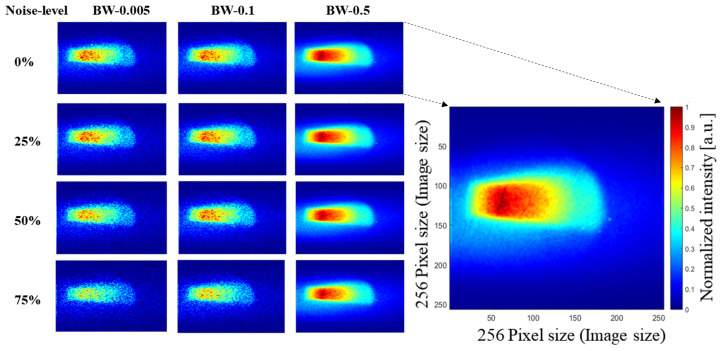
Examples from our dataset illustrating four different noise levels and three different bin widths (BW-0.005, BW-0.1, BW-0.5).

To achieve optimal denoising results, we initiated our model training by utilizing the official pre-trained weights of SCUNet. Subsequently, we fine-tuned the model on our specific dataset, enhancing its adaptability to our unique denoising requirements. During this training process, a compound loss function was employed to synergistically improve the overall performance of the model. Figure 4 shows examples of images in our dataset with varying noise levels and bin widths. In the first row, we present our original data with 100% of the initial number of photons. Subsequently, we reduce the number of photons by a factor of 25% in each following row, simultaneously increasing the sampling rate to 0.05, 0.1, and 0.5. In this figure, the highest quality image is the one with a noise level of 0% and maximum bin width (BW) of 0.5 (see first row and third column), and the lowest quality image is the one with a 75% noise level and bin width of 0.005. An enlarged image is shown in this figure for clarity, having an image size of 256 × 256 pixels.

**Figure 5:**
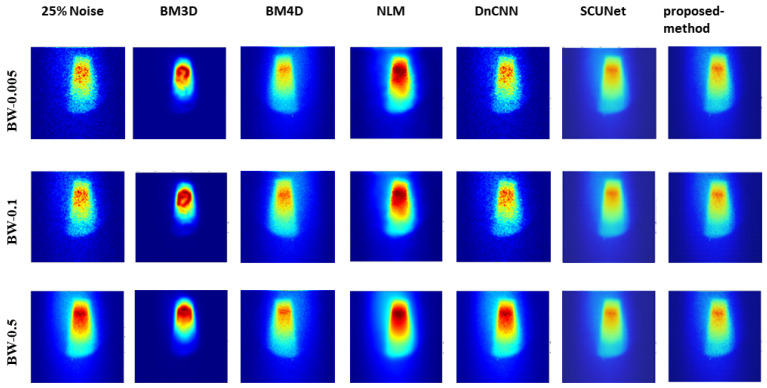
Visual comparison: predicted results of our method alongside those of other methods (noise level 25%).

**Figure 6:**
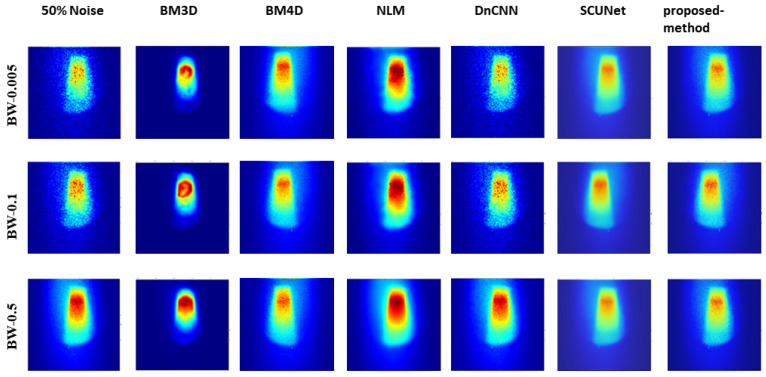
Visual comparison: predicted results of our method alongside those of other methods (noise level 50%).

**Figure 7:**
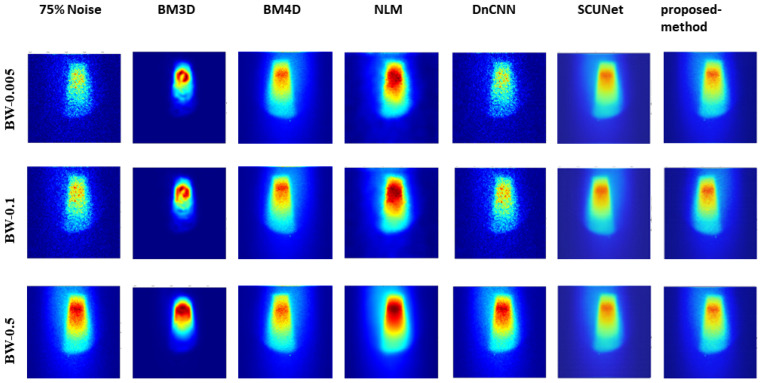
Visual comparison: predicted results of our method alongside those of other methods (noise level 75%).

Figure 5, Figure 6, and Figure 7 show the predicted denoising qualitative results for various methods at noise levels of 25%, 50%, and 75%, respectively. These tables compare state-of-the-art techniques with our proposed model on our dataset. Results for the BM3D method are presented in the second row of each table. BM3D, a 3D block-matching algorithm primarily used for noise reduction in images, is an extension of the NLM methodology [47]. In the third column, the results for an enhanced version of MD3D are illustrated, which integrates a combination of BM4D within the 3D shearlet transform realm, alongside a generative adversarial network, for image denoising [31]. The fourth column displays the outcomes of the NLM method, which involves calculating the mean value for all pixels in the images, with weights assigned based on the similarity of each pixel to the target pixel [48]. Moving to the fifth column, we observe the results for the DnCNN [49]. This method aims to recover the clean image *x* from the noisy image y=x+v, assuming *v* is additive white Gaussian noise (AWGN). The network can handle Gaussian denoising with an unknown noise level. In general, image denoising methods can be categorized into two major groups: model-based methods, like BM3D, which are flexible in handling denoising problems with various noise levels but are time-consuming and require complex priors, and discriminative learning-based methods, like DnCNN, developed to overcome these drawbacks. In the sixth column, we present the results for blind image denoising via the SCUNet method [50], while existing methods rely on simple noise assumptions, such as AWGN, the SCUNet method is designed to address all unknown noise types that remained unsolved in previous methods. The last column shows the results of our method, which extends the performance of the SCUNet method. The qualitative results of the proposed model consistently yield superior visual outcomes across all different bin widths, while BM3D exhibits better visual results, it tends to produce smoother images, consequently removing important details. In contrast, the proposed method generates images that closely resemble the original (0% noise level) image. Conversely, DnCNN produces results that closely match images at the same noise level.

**Figure 8:**
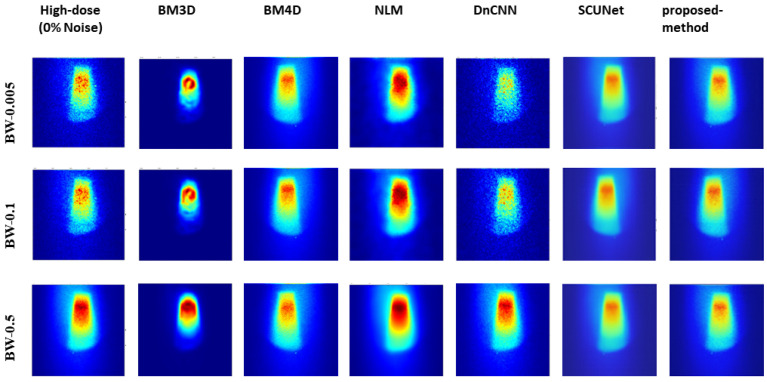
Visual comparison: predicted results of our method alongside those of other methods with the original image (noise level 0%).

Figure 8 presents the comparison results of predicted denoising for various methods at a 75% noise level with reference to the 0% noise condition. The findings indicate that our proposed model exhibits significantly improved visual results, closely resembling the original image even under high and low sampling rates.

In addition to the qualitative comparison of different methods, we present the quantitative results for noise levels of 25%, 50%, and 75%, based on two measurement factors, PSNR and SSIM, respectively, in Table 1 and Table 2. The compared methods are BM3D, BM4D, NLM, DnCNN, and SCUNet. It can be observed that our proposed method achieved significantly better PSNR and SSIM results than other methods for the highest noise levels. Except in comparison with BM3D and DnCNN, which have in some cases higher PSNR and SSIM for the two noise levels of 25% and 50%, our method outperformed the others in terms of PSNR and SSIM. However, possible reasons for the better performance of DnCNN could be because of its match with the same noise level. We can see from the results of both tables that the SCUNet method achieves lower quantitative metrics (i.e., PSNR and SSIM) at all noise levels compared to our proposed model. The computational time performance for other methods, such as the SCUnet model, is around 0.072 s, whereas the computational time for BM4D inference is around 0.064 s, indicating a negligible increase in performance. The proposed model’s computational time is at around 0.059 s. Thus, the time performances of these methods are roughly on the same order magnitude.

## 4. Discussion and Conclusions

In this study, we refined the SCUNet deep learning model to enhance its applicability for background noise reduction in XFCT images. To optimize the application to our image data, we implemented a compound loss function [51] aimed at capturing shape-aware weight maps, addressing specific challenges posed by medical images within the SCUNet architecture. Our approach, as indicated in Table 1 and Table 2, demonstrated improved outcomes, showcasing the effectiveness of the compound loss function [51].

To assess the efficacy of our approach, we conducted comparative analyses against five existing models BM3D, BM4D, NLM, DnCNN, and the original SCUNet. These comparisons demonstrate the efficiency of our blind deep learning model in eliminating all types of noise, including unknown noise, alongside known image noise such as AGN. Our overarching goal is to produce clean XFCT images from concurrent noisy images, corresponding to low-dose image acquisition, in order to enable low-dose XFCT imaging.

To facilitate our research, we curated a dataset comprising reconstructed images derived from sparse-view benchtop XFCT images. The deep learning model underwent training and evaluation, utilizing two key metrics, PSNR and SSIM. The results on experimental data provide insights into the effectiveness of our proposed method for denoising Gaussian noise and suggest the potential practicality of the trained deep blind model in handling real noisy images. In this study, we only present the preliminary results of our proposed method due to the constraints imposed by the limited availability of training data.

Analysis of the results, as illustrated in Figure 8, shows the robust performance of our model across varying noise levels and low-dose scenarios, transcending three different bin widths. Conversely, other methods exhibited performance dependency on bin width values and noise levels. Further insights from Figure 5, Figure 6, and Figure 7 showcased the output of all models under different noise levels of 25%, 50%, and 75%, respectively.

Quantitative assessments of noise removal demonstrated better performance of our proposed model compared to BM3D, NLM, and the original SCUNet. Our method demonstrated notable proficiency in handling images with high noise levels and sparse sampling, surpassing other studied methods, including SCUNet. Finally, our method achieved a maximum PSNR of 39.05 and SSIM of 0.86, indicating the improved performance of our model. We believe this framework offers a strong foundation for further optimization and tailoring of the SCUNet model specifically for the complexities of XFCT image denoising. However, our study has several limitations, primarily arising from the limited XFCT data available for model training. The presented results consider data and augmented samples from only a single phantom shape and size, corresponding to the small-animal-size scale. These phantoms are homogeneous water-filled borosilicate containers with hexagonal outer shapes, embedding contrast-filled targets. Additionally, we only have data corresponding to a single contrast agent (i.e., gadolinium at varying concentrations) and target size (i.e., around 8 mm diameter polypropylene microcentrifuge tubes filled with Gd). Correspondingly, due to the limited availability of training and reference data, unlike established anatomical imaging methods like CT and MRI, our present study has limits in comprehensively identifying potential artifacts and describing the detailed impact of our denoising model on the shape, size, and other properties of both target and background features. To improve upon current limitations and extend the model’s capabilities, our future work could investigate the proposed method on a wider range of X-ray fluorescence data generated using Monte Carlo simulations. This data could encompass a variety of target and background shapes, sizes, and features, as well as different nanoparticle-based contrast agents. This would also enable us to investigate the various influencing factors, physical or algorithmic, affecting image quality and overall noise removal performance.

## Figures and Tables

**Table 1:** Quantitative results of the various denoising methods based on PSNR.

Bin Widths	Noise Level	BM3D	BM4D	NLM	DnCNN	SCUNet	Proposed Model
BW-0.05	25%	13.93	31.44	27.92	36.82	22.77	29.68
	50%	13.91	28.61	25.16	31.07	24.51	26.97
	75%	13.89	29.18	24.58	24.75	25.29	31.44
BW-0.1	25%	13.70	29.78	32.42	38.88	22.87	35.48
	50%	13.70	31.20	28.19	34.50	27.82	29.06
	75%	13.67	27.02	27.71	27.75	26.77	29.33
BW-0.5	25%	11.76	28.18	39.94	49.35	22.54	31.81
	50%	11.75	33.27	38.77	43.66	22.74	32.67
	75%	11.75	31.22	36.75	38.40	25.58	39.05

**Table 2:** Quantitative results of the various denoising methods based on SSIM.

Bin Widths	Noise Level	BM3D	BM4D	NLM	DnCNN	SCUNet	Proposed Model
BW-0.05	25%	0.073	0.7902	0.7435	0.9430	0.4661	0.7868
	50%	0.0720	0.7710	0.739	0.872	0.5177	0.8369
	75%	0.071	0.7917	0.7156	0.7431	0.6472	0.8654
BW-0.1	25%	0.0716	0.7425	0.7985	0.8594	0.6153	0.8284
	50%	0.0715	0.7154	0.7984	0.8023	0.6206	0.8658
	75%	0.0712	0.7556	0.7801	0.7867	0.6453	0.8218
BW-0.5	25%	0.0617	0.8349	0.8029	0.9139	0.5773	0.8786
	50%	0.0616	0.8314	0.7626	0.9014	0.4972	0.8466
	75%	0.0615	0.8424	0.7611	0.7582	0.5290	0.8638

## Data Availability

The dataset used within this study can be obtained here: https://zenodo.org/records/11243130 (accessed on 7 March 2024). All other related raw data and datasets supporting the results and conclusions of this article will be made available by the authors without undue reservations.

## Abbreviations

The following abbreviations are used in this manuscript:

XFCT	X-ray fluorescence computed tomography
XRF	X-ray fluorescence
AI	Artificial Intelligence
DL	Deep Learning
SCUNet	Swin-Conv-UNet
CT	Computed Tomography
PSNR	Peak signal-to-noise ratio
SSIM	Structural Similarity Index
BM3D	Block-Matching and 3D filtering
BM4D	Block-Matching and 3D filtering
MSE	Mean Squared Error
NLM	Non-local means
DnCNN	Denoising Convolutional Neural Network
CNN	Convolutional Neural Network
GAN	Generative Adversarial Networks
Gd	Gadolinium
wt	Weight
Swin	Shifted window Transformer
keV	Kilo electron volt
mA	milliampere
CBCT	Cone-Beam Computed Tomography
MAP	Maximum A Posteriori
DRUNet	Dilated-Residual U-Net
SwinIR	Image Restoration Using Swin Transformer
SConv	Strided Convolution
TConv	Transposed Convolution
RConv	Residual Convolutional
SwinT	Swin Transformer
BW	Bin width
AWGN	Additive White Gaussian Noise
